# Can angiogenesis inhibitor therapy cause changes in imaging features of hepatic hemangioma- Initial study

**DOI:** 10.3389/fonc.2023.1134179

**Published:** 2023-03-10

**Authors:** Tang Liu, Wenxue Pan, Shengyuan Lai, Jiawen Luo

**Affiliations:** Department of Radiology, The Second Affiliated Hospital of Dalian Medical University, Dalian, Liaoning, China

**Keywords:** angiogenesis inhibitor, vascular endothelial growth factor, bevacizumab, hemangioma, tumor, computed tomography

## Abstract

**Background:**

To observe whether anti-angiogenesis therapy can induce changes in size and enhancement characteristics of hepatic hemangioma.

**Method:**

133 patients with hepatic hemangioma lesions were analyzed and classified into a Bevacizumab group (n=65) and the control group (n=68). The parameters (Volume, CT enhancement ratio, enhancement patterns) of pre-and post-treatment in the bevacizumab and control groups independently calculated and compared by two radiologists. Correlation among the systolic blood pressure, diastolic blood pressure, heart rate with the hemangioma volume was evaluated using Pearson’s correlation analysis.

**Results:**

The hepatic hemangioma volume was significantly decreased after treatment in the Bevacizumab group (8.6 ± 18.7mL *vs*.7.3 ± 16.3mL, P<0.05), and there was no significant change in the control group (15.1 ± 19.8mL *vs*.15.4 ± 20.7mL, P = 0.504). A significant difference in enhancement patterns of hepatic hemangiomas was observed after treatment with Bevacizumab (P<0.01). There was no significant difference in arterial phase (AP)enhancement rate and arterial phase-portal venous phase (AP-PVP) enhancement ratios after treatment in the Bevacizumab and control groups (Ps>0.05).The Pearson correlation results showed that blood pressure, heart rate, and hemangioma volume were unrelated or weakly related before and after bevacizumab treatment under the control of factors including weight, contrast injection scheme and CT scanning scheme.

**Conclusions:**

Anti-angiogenesis therapy can cause changes in enhancement pattern and volume of hepatic hemangioma. Radiologists should pay more attention to the reexamination of tumor patients treated with anti-angiogenesis therapy.

## 1 Introduction

Over the years, angiogenesis inhibitors have been widely used to treat cancer and eye disorders (1, 2). Vascular endothelial growth factor(VEGF) is highly expressed in malignant cells. It has been established that VEGF can stimulate the proliferation and survival of endothelial cells and lead to the formation of new blood vessels, which is an effective therapeutic target for anticancer treatments (3–5). As a recombinant anti-VEGF antibody, Bevacizumab can inhibit the binding of all VEGF isoforms to VEGFR-1 and VEGFR-2 receptors, reduce angiogenesis and inhibit tumor growth (6). Bevacizumab is often used in combination with chemotherapy for the treatment of colon cancer, lung cancer, glioblastoma, and kidney cancer (4).Bevacizumab combined with 5-fluorouracil (5‐FU) and leucovorin (LV) is well-established to be more effective in the treatment of metastatic colorectal cancer than 5‐FU‐LV association alone (2).

Hepatic hemangioma is the most common benign tumor, with an incidence rate of up to 20%, ranging from a few millimeters to more than 20cm (7–10). Hepatic hemangioma is more frequent in females and common in the right lobe of the liver (11, 12). Most patients with hepatic hemangioma have no obvious symptoms, and most cases are incidental findings during imaging examinations. It is widely acknowledged that the typical imaging diagnosis of hepatic hemangioma can replace pathological diagnosis (13). Interestingly, shrinkage of hepatic hemangioma after angiogenesis inhibitor therapy has been observed in some studies. Yamashita et al. found that sorafenib could effectively reduce the size of giant cavernous hepatic hemangioma (14). Mahajan et al. found that liver hemangioma shrunk following treatment with Bevacizumab (15). However, Lee et al. found that hemangiomas did not shrink in patients receiving Bevacizumab for colorectal cancer and sunitinib for renal cell carcinoma (16).Although most studies have found that bevacizumab can reduce the size of liver hemangiomas, some studies have found that the size does not change. However, most of these studies are based on case reports and small sample data, so the results are confusing.

In clinical work, we observed that hepatic hemangioma exhibit changes in enhancement pattern after treatment with bevacizumab, such as a shift from progressive enhancement to circular enhancement or continuous enhancement to insignificant enhancement, which is difficult to distinguish from liver metastases, liver lymphoma and other liver tumors (17).Therefore, we designed a study on the changes of hemangioma volume, enhancement pattern and enhancement rate after bevacizumab treatment, that based on large samples and statistical analysis. The purpose of this study was to observe whether anti-angiogenesis therapy can induce changes in volume and enhancement patterns of hepatic hemangioma.

## 2 Materials and methods

### 2.1 Ethical considerations

This study was approved by the Second Affiliated Hospital of Dalian Medical University Ethics Committee.

### 2.2 Patients

We collected the patients with malignant tumor and hemangioma admitted to our hospital from November 2015 to June 2022.All patients underwent enhanced multiphase CT of the upper abdomen, and their blood pressure and heart rate were recorded during the CT examination. All CT images were independently and blindly viewed by two radiologists (15 and 10 years of expertise in abdominal radiology, respectively). All patients underwent triple-phase contrast-enhanced scanning (arterial phase, portal venous phase and delayed phase). Two radiologists assessed whether each imaging phase was accurate based on the criteria of CT/MRI LI-RADS ^®^ V2018. The specific evaluation indicators are provided in **Supplementary Material 1** (18). The two radiologists judged the enhancement patterns, divided into five types (progressive, continuous, circular, insignificant and delayed enhancement). A detailed judgment index is provided in **Supplementary Material 2** (17, 19–21). Disagreements were resolved by consensus-based discussion. The two typical enhancement patterns of hepatic hemangioma are progressive and continuous enhancement.

The inclusion criteria for the Bevacizumab group consisted of patients who received chemotherapy and bevacizumab treatment for more than 3 cycles (each cycle lasting approximately 21 days). Contrast-enhanced abdominal CT scans were performed within 2 weeks before and after bevacizumab treatment. The exclusion criteria for the Bevacizumab group consisted of (1) Poor image quality or (2) Patients with previous liver surgery or TACE (transcatheter arterial chemoembolization) or(3)Two or more anti-angiogenic agents.

The inclusion criteria of the control group consisted of patients that only received chemotherapy for more than 3 cycles but did not receive bevacizumab treatment. Contrast-enhanced abdominal CT scans were performed within 2 weeks before and after chemotherapy. The exclusion criteria for the control group consisted of (1) Poor image quality or (2) Patients with previous liver surgery or TACE.

**Figure 1:**
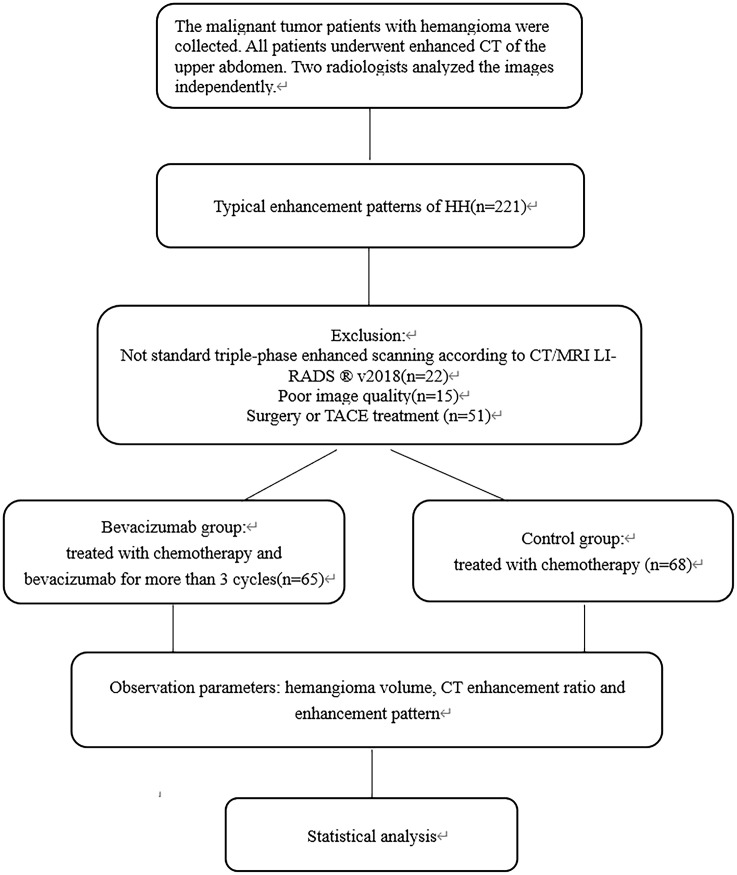
Flow chart of Bevacizumab group and control group.

A total of 221 malignant tumor patients with a typical enhancement pattern of hemangioma judged by two experts were selected, of which 88 cases were excluded (Not standard triple-phase enhanced scanning (n=22), Poor image quality(n=15), Surgery or TACE treatment (n=51)).The final study population included 133 patients, including 65 patients in the bevacizumab group and 68 patients in the control group. The flowchart for patient selection is shown in **Figure 1**. The bevacizumab group comprised 33 women and 32 men (age range, 42-80 years; mean age, 61.8 years) treated with Bevacizumab (400mg per day) for an average of 6.7 cycles (Range 3-32 cycles), with each cycle lasting for 21 days. The control group comprised 34 women and 34 men (age range, 40-77 years; mean age, 59.2 years).

### 2.3 Imaging technique

All patients underwent contrast-enhanced abdomen CT examinations using a Philips brilliance ICT 256 (Philips Healthcare, Netherlands). Detailed imaging parameters are described in **Table 1**. Non-ionic contrast material (iohexol, Yangzi River Pharmaceutical, China) with 1 ml/kg of body weight was injected intravenously at 2.0-3.0 ml/s. Bolus tracking was applied, and the arterial phase (AP) scan was initiated from an aortic enhancement of 150 HU. The portal venous phase (PVP) scan was initiated 40 seconds after the start of AP. The delayed phase (DP) scan was initiated 120 seconds after the start of AP. The patient’s position was supine in all phases. All CT datasets were transferred to Philips post-processing workstation (EBW, Extended Brilliance Workspace).

**Table 1:** CT Imaging Parameters.

Voltage	current	width of the collimator	Matrix	Spiral factor	Layer thickness
120 kVp	220 mA	128 × 0. 625 mm	512×512	0.8	1mm

### 2.4 Imaging analysis

Two radiologists who were blinded to the clinical information of the patient (with 15 and 10 years of experience in interpreting abdominal CT, respectively) evaluated the CT images retrospectively in consensus on the Philips post-processing workstation (EBW). All parameters (volume and the CT value of each phase)of each lesion before and after treatment were measured three times, and their average values were calculated for future statistical analysis to reduce the effect of different region of interest (ROI) delineation and measurement by different observers.

### 2.5 Hemangioma volume

The volume measurement was performed on the portal phase using EBW. First, the semi-automatic function of the software was used to mark the lesion boundary, and the lesion boundary was modified and examined layer by layer in combination with the cross-section, coronal plane and sagittal plane. Moreover, the labeled lesions were stained, and the volume of the lesion was calculated using the volume calculation software of the workstation.

### 2.6 Enhancement patterns

We used the consensus interpretation of the two radiologists to determine the enhancement patterns, divided into progressive, continuous, circular, insignificant and delayed enhancement.

### 2.7 CT enhancement ratio

All CT values of each lesion before and after treatment were measured at the same imaging level of lesions in unenhanced scans, AP and PVP. The ROI was drawn along the lesion margin by two experienced radiologists independently. CT values were evaluated 3 times, and their average values were calculated. The enhancement ratios were calculated according to formula(1-2) (22):


(1) $$AP\;enhancement\;ratio=\left(CT\;value_{AP}\;-\;CT\;value_{\;unenhanced\;scan}\right)/CT\;value{\;}_{unenhanced\;scan}$$

(2) $$\begin{array}{c} AP-PVP\;enhancement\;ratio=\left(\;CT\;value_{PVP}-\;CT\;value_{\;unenhanced\;scan}\right)\\ /\left(CT\;value{\;}_{AP}\;-\;CT\;value{\;}_{unenhanced\;scan}\right) \end{array}$$

### 2.8 Statistical analysis

All data analyses were performed using standard software (SPSS v17.0 for Windows; SPSS Inc., Chicago, IL, USA) and R language. Intraclass correlation coefficient (ICC) was used to evaluate the consistency of image judgment between the two observers. The degree of agreement was categorized as follows: 0.00 to 0.39, poor agreement; 0.40 to 0.59, fair agreement; 0.60 to 0.74, good agreement; and 0.75 to 1.00, excellent agreement (23). The quantitative data were demonstrated as mean ± standard deviation (SD), and the qualitative data were presented as frequencies. A t-test was used to compare intra-group pre-and post-treatment differences. The χ2test was used to compare the difference in enhancement pattern between pre-and post-treatment. Correlation among blood pressure, heart rate with the hemangioma volume was evaluated using Pearson’s correlation analysis. All P-value< 0.05 was statistically significant.

## 3 Results

### 3.1 Clinical information of patients

There were no significant differences in age, sex, primary tumor between Bevacizumab and control group (P > 0.05) (**Table 2**).

**Table 2:** Comparison of general conditions between the bevacizumab group and control group.

group	n	Sex male/female	age years (median)	Primarytumor colorectal/lung/stomach cancer
Bevacizumab	65	32/33	61.8	31/23/11
Control	68	34/34	59.2	25/23/20
*P*		1	0.053	0.203

P< 0.05 was statistically significant.

### 3.2 Intraclass correlation coefficient

The two radiologists exhibited excellent agreement in observing the above parameters (All ICC values> 0.9). Therefore, the measurement data from the two observers are suitable for the following analysis.

### 3.3 Hemangioma volume

**Figure 2:**
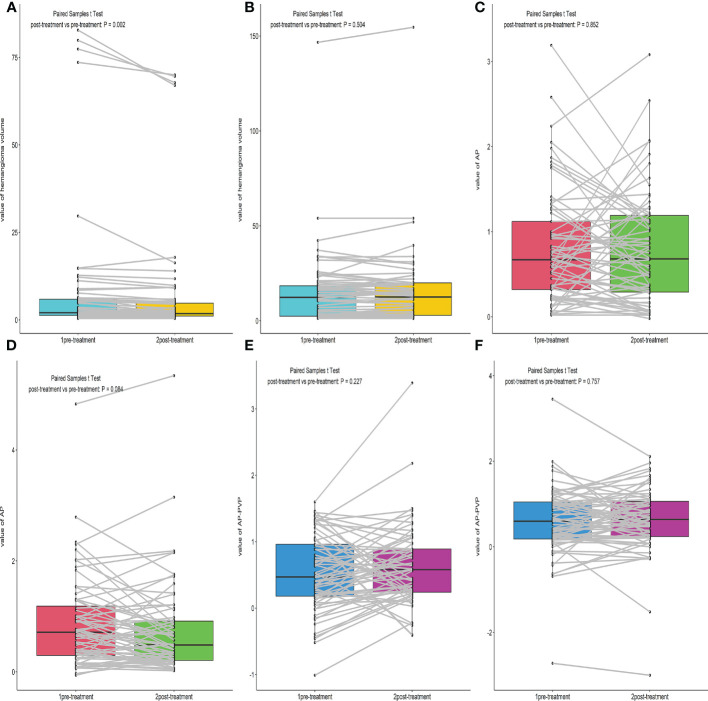
Box plot of the Hemangioma volume, AP, AP-PVP before and after treatment. **(A, B)** Comparison of hemangioma volume before and after treatment of the Bevacizumab and Control group. **(C–F)** Comparison of hemangioma AP, AP-PVP before and after treatment of the Bevacizumab and Control group.

**Figure 3:**
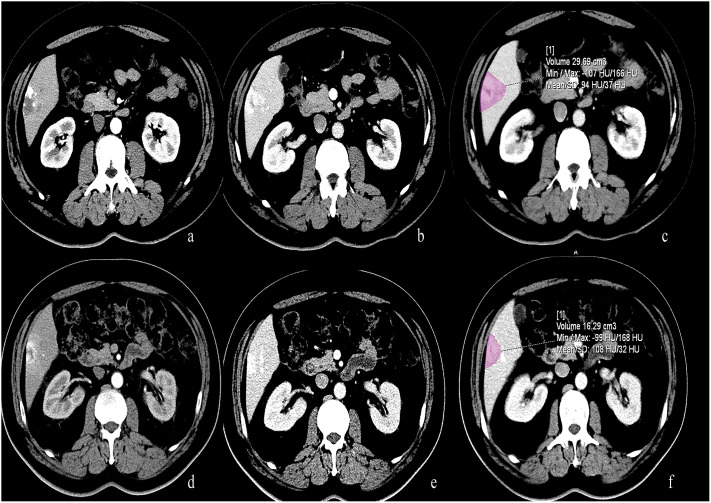
50-year-old male with lung cancer who received 5 cycles of treatment with bevacizumab. **(A, B)** Pre-treatment CT scan showed a typical hemangioma located at the liver segment 6. **(C)** The volume of this hemangioma measured 29.69 ml. **(D–F)** After treatment with Bevacizumab, the volume of this hemangioma decreased and measured 16.29 ml.

We compared the hemangioma volume before and after treatment (**Figures 2A, B**, **3**). A statistically significant difference in the volume of the liver hemangiomas (P< 0.05) was observed between pre-treatment CT (range 0.4–82.8 mL; mean ± SD 8.6 ± 18.7mL) and post-treatment CT (range 0.4–70 mL; mean ± SD 7.3 ± 16.3mL) images in the Bevacizumab group. No significant difference in the volume of the liver hemangiomas was observed between pre-treatment (range 1.2–146.7mL; mean ± SD 15.1 ± 19.8mL) and post-treatment CT (range 1.18–154.6mL; mean ± SD 15.4 ± 20.7mL) images in the control group (P = 0.504).

### 3.4 Enhancement patterns

**Figure 4:**
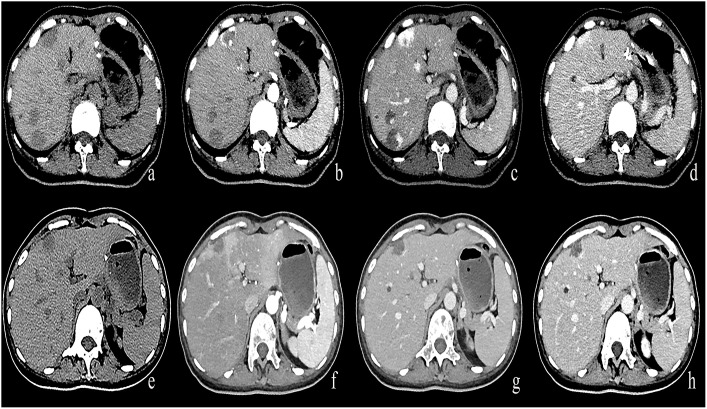
**(A–D)** A round low-density mass in the left inner lobe of the liver was shown in the plain CT. The lesions demonstrate peripheral nodular discontinuous enhancement in the arterial phase and centripetal filling in the portal and delayed phases. **(E–H)** After treatment with Bevacizumab, the lesions showed insignificant enhancement.

**Figure 5:**
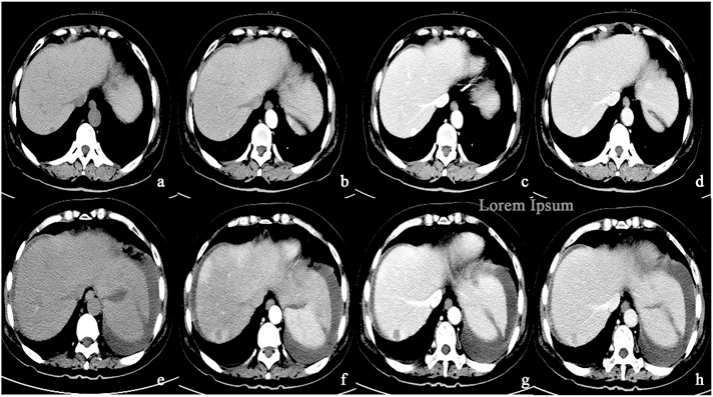
**(A–D)** A round low-density mass in the right lobe of the liver was shown in the plain CT. The lesions demonstrate peripheral nodular discontinuous enhancement in the arterial phase and centripetal filling in the portal and delayed phases. **(E–H)** After treatment with Bevacizumab, the lesions showed circular enhancement.

In the Bevacizumab group, there were 43 cases of progressive enhancement and 22 cases of continuous enhancement before treatment. After treatment, the enhancement mode of some lesions changed as follows, from progressive to circular enhancement (n=2), from progressive to insignificant enhancement (n=4), from continuous to circular enhancement (n=1), and the remaining cases exhibited no change in enhancement mode (n=58). There was a statistically significant difference (P< 0.01) in enhancement patterns between pre-and post-treatment of the Bevacizumab group(**Figures 4**, **5**).

In the control group, there were 56 cases of progressive enhancement and 12 cases of continuous enhancement before treatment. There was no change in the enhancement pattern after treatment.

### 3.5 CT enhancement ratio

There was no significant difference in AP enhancement rate and AP-PVP enhancement ratio between pre-and post-treatment of Bevacizumab and control groups (Ps> 0.05) (**Figures 2C–F**).

Correlation analysis between blood pressure, heart rate and hemangioma volume

**Figure 6:**
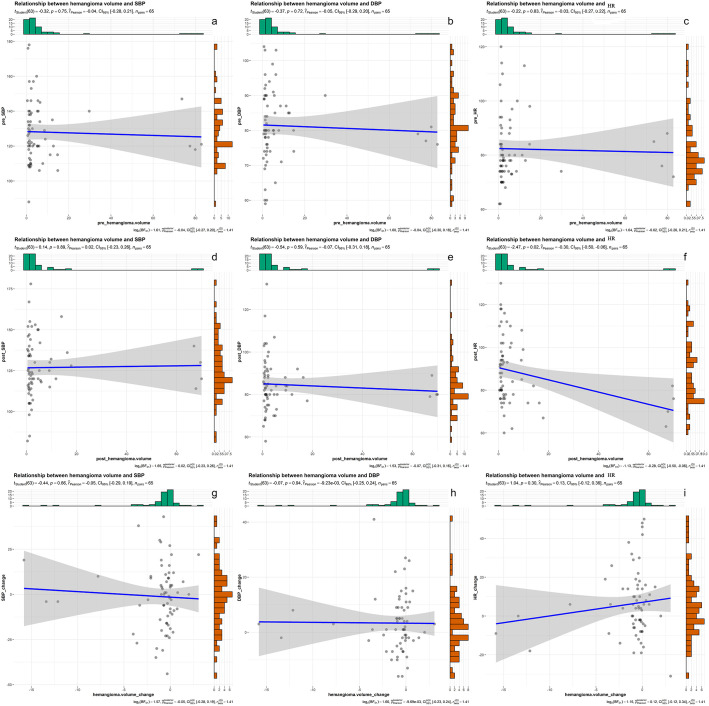
**(A–C)** the correlation results of systolic blood pressure, diastolic blood pressure, heart rate and hemangioma volume before bevacizumab treatment. **(D–F)** the correlation results of systolic blood pressure, diastolic blood pressure, heart rate and hemangioma volume after bevacizumab treatment. **(G–I)** the correlation results of changes in systolic blood pressure, diastolic blood pressure, heart rate and changes in hemangioma volume after bevacizumab treatment.

As shown in **Figure 6**, there was a weak negative correlation between heart rate and hemangioma volume after bevacizumab treatment (r = -0.3, P< 0.05). There was no correlation between heart rate and hemangioma volume before bevacizumab treatment. And hemangioma volume did not correlate with Systolic and diastolic blood pressure.

## 4 Discussion

Hepatic hemangioma is the most common benign hepatic tumor. The two typical enhancement patterns of hepatic hemangioma are progressive and continuous enhancement, which is considered to replace pathological diagnosis. In clinical work, we found that in some tumor patients with hepatic hemangioma, the volume and enhancement pattern of the hemangioma changed after antiangiogenic therapy. After consulting the literature, we found that many research results showed that anti-angiogenic drugs such as Bevacizumab could reduce the size of hemangioma during the treatment of tumors. However, most of these studies are based on case reports and small sample data. Therefore, we designed a study based on large samples and statistical analysis to observe the changes in the volume, enhancement pattern and enhancement rate of hemangioma after treatment with bevacizumab, and observe the effects of blood pressure and heart ratio on the results. Our results showed a significant difference in the volume and enhancement pattern of hemangiomas in the Bevacizumab group after treatment. There is such a scenario that patients often lack previous imaging data when they seek treatment at different medical institutions. It is difficult to distinguish hepatic hemangiomas causing changes in imaging features from hepatic metastases, lymphoma, and other liver tumors, which confuses imaging physicians in diagnosis and also leads to confusion in establishing clinical treatment plans. Therefore, imaging physicians should raise their awareness of this situation. In our experience, when it is difficult to distinguish hemangiomas with altered enhancement mode from other liver tumors, especially liver metastases, we can use the following methods to differentiate them: First of all, we must pay attention to the acquisition of the patient’s previous imaging data. Second, although some of the liver hemangioma in circular enhancement, but there are still some differences with liver metastases, although liver metastases also showed circular enhancement, their enhancement ring thickness is thicker, and the central density is lower, showing halo sign and bull eye sign. Finally, the ADC value of magnetic resonance is helpful for distinguishing hepatic hemangioma from metastatic tumor (24). The ADC value of hepatic hemangioma is higher, while that of liver metastases is lower.

Our results are consistent with most studies. Kanemura et al. documented a case of epithelioid hemangioendothelioma (EHE) originating from the pleura and treated with carboplatin, pemetrexed and Bevacizumab, leading to an obvious reduction in symptoms (25). A study found that capecitabine combined with Bevacizumab was more effective in treating metastatic hepatic EHE than capecitabine alone (26). Yeo et al. documented a case of recurrent multiple nonresectable intracranial hemangiomas treated with Bevacizumab and alkylating agent temozolomide. After 2 months of treatment, the tumor size decreased by 30%, completely degenerated at 6 months, and continued to degenerate 36 months after cessation of therapy (27). Moreover, it has been reported that the size of recurrent sinonasal hemangioma treated with intralesional bevacizumab injection decreased by 50% within 1 month after injection (28).Consistent with most study results, we found that the volume of hemangiomas in the Bevacizumab group significantly decreased after treatment. On the other hand, Lee et al. substantiated that hemangiomas in patients who received Bevacizumab for colorectal cancer and sunitinib for renal cell carcinoma did not shrink (16). Compared with Lee’ study, we are convinced that our results are more reliable for the following three reasons. First of all, the sample size of our study was relatively large, and the study results were based on statistical analysis. Moreover, we used the semi-automatic function of the software to mark the lesion boundary and calculate tumor volume to avoid manual error. Finally, we designed a control group to verify the results; our findings showed no significant difference in the hemangioma volume after treatment in the control group.

Hepatic hemangioma is a mesenchymal lesion consisting of blood-filled vascular spaces of different sizes, surrounded by a simple layer of endothelial cells and supported by fibrous connective tissue (7, 29). Given that the typical enhancement characteristics of hemangioma are related to its unique histology when the contrast material is injected, the vascular space is gradually and slowly filled with iodine-laden blood, thus accounting for progressive or continuous enhancement characteristics. Our results show that bevacizumab treatment can change the enhancement pattern of hemangioma. We speculate that the reason may be that bevacizumab therapy inhibits the proliferation of endothelial cells and reduces the micro-vessel density of tumors, thereby changing the histological composition of hepatic hemangioma, and ultimately leading to atypical imaging manifestations of hemangioma.

There is ample evidence that the tumor enhancement ratio is positively correlated with the tumor micro-vessel density (MVD) (30, 31). The AP enhancement rate reflects the density of the neovascularization in the tumor, while the AP-PVP enhancement ratio reflects the permeability of the neovascularization (22). Since we established that Bevacizumab could reduce tumor angiogenesis, we speculate that after bevacizumab treatment, the CT enhancement ratio of hepatic hemangioma will be reduced. Nonetheless, our results showed no significant difference in AP enhancement rate and AP-PVP enhancement ratio between pre-and post-treatment values in the Bevacizumab group. Indeed, larger sample sizes are warranted for our future studies to increase the robustness of our findings.

It has been reported that cardiac output and cardiovascular circulation time may affect the accumulation time of the contrast agent. Low blood volume or high blood pressure may lead to decreased the clearance rate of the contrast agent, resulting in an increase or delay in peak accumulation of the contrast agent (32).Our results showed that blood pressure, heart rate, and hemangioma volume were unrelated or weakly related before and after bevacizumab treatment under control of weight factors, contrast injection scheme and CT scanning scheme. It is suggested that the study of hemangioma volume change is not affected by blood pressure and heart rate. Because of the difference in sample size between the two groups (unchanged and changed enhancement mode), the influence of blood pressure and heart rate on the enhancement pattern was further investigated after the sample size was increased.

Our study has several limitations. First, this is a single-center retrospective study. The size of our study population was not sufficiently large, consisting entirely of adults without infants or children, which is not enough to generalize our findings. Besides, our study population consisted of patients carrying incidental asymptomatic hemangiomas. Hemangiomas are usually small and harbor the possibility of measurement error. Moreover, since these incidental hemangiomas were not surgically removed, we could not obtain pathological confirmation. However, given that the imaging diagnosis of hepatic hemangioma is widely accepted, we strictly applied imaging criteria to exclude all atypical lesions. Finally, our study population consisted of patients with multiple tumors, not a single tumor. However, the chemotherapy schemes for different tumors are different. Indeed, due to the complexity of drug interactions, this unknown drug interaction may have some impact on our research results. In the future studies, the sample size will be increased, and the patients divided into groups according to their primary tumors to improve the accuracy and reliability of our findings. In addition, in the present research, the study population consisted of tumor patients treated with Bevacizumab for more than three cycles. Stratifying based on the number of cycles can be conducted in future studies to assess its effect on the efficacy of Bevacizumab. Finally, magnetic resonance imaging is less because reexamination of tumor patients is based mainly on enhanced CT. Whether the MR characteristics of hepatic hemangioma change after antiangiogenic therapy should be further investigated.

## 5 Conclusion

Anti-angiogenesis therapy can cause changes in enhancement pattern and volume of hepatic hemangioma. Radiologists should pay more attention to the reexamination of tumor patients treated with anti-angiogenesis therapy.

## Data availability statement

The raw data supporting the conclusions of this article will be made available by the authors, without undue reservation.

## Ethics statement

The studies involving human participants were reviewed and approved by the Second Affiliated Hospital of Dalian Medical University Ethics Committee. The patients/participants provided their written informed consent to participate in this study.

## Author contributions

TL, WP, SL, JL participated in the conception and designed this study. TL, WP participated in the collection and arrangement of research data. SL, JL completed the analysis and interpretation of the data. TL wrote the manuscript. All authors contributed to this article and approved the submitted version.
