# Supplementary material for: Can angiogenesis inhibitor therapy cause changes in imaging features of hepatic hemangioma- Initial study
Source: Front Oncol. 2023 Mar 10;13:1134179. doi: 10.3389/fonc.2023.1134179 (PMC10036792; doi:10.3389/fonc.2023.1134179)
Supplement: Supplementary file 1 [file Table_1.docx]

Supplementary Material

Can angiogenesis inhibitor therapy cause changes in imaging features of hepatic hemangioma- Initial study

Tang Liu* , Wenxue Pan*, Shengyuan Lai

* Correspondence: Jiawen Luo: [kaoyan2006succeed@163.com](mailto:kaoyan2006succeed@163.com)

Supplementary Material 1: Phases of enhancement (CT/MRI LI-RADS ® v2018 CORE)

| phases | the following characteristics |
| --- | --- |
| Arterial phase (AP)  Late AP | •Hepatic artery and branches are fully enhanced  • Hepatic veins not yet enhanced by antegrade flow  • Subtype of AP in which portal vein is enhanced |
| Portal venous phase  (PVP) | • Portal veins are fully enhanced  • Hepatic veins are enhanced by antegrade flow  • Liver parenchyma usually is at peak enhancement |
| Delayed phase  (DP) | • Portal and hepatic veins are enhanced but less than in PVP  • Liver parenchyma is enhanced but usually less than in PVP  Typically acquired 2 to 5 minutes after injection |
