# Supplementary material for: Can angiogenesis inhibitor therapy cause changes in imaging features of hepatic hemangioma- Initial study
Source: Front Oncol. 2023 Mar 10;13:1134179. doi: 10.3389/fonc.2023.1134179 (PMC10036792; doi:10.3389/fonc.2023.1134179)
Supplement: Supplementary file 2 [file Table_2.docx]

Supplementary Material

Can angiogenesis inhibitor therapy cause changes in imaging features of hepatic hemangioma- Initial study

Tang Liu* , Wenxue Pan*, Shengyuan Lai

* Correspondence: Jiawen Luo: [kaoyan2006succeed@163.com](mailto:kaoyan2006succeed@163.com)

Supplementary Material 2: Patterns of contrast enhancement on CT

| Enhancement type | Detailed judgment index |
| --- | --- |
| Progressive | lesions demonstrate peripheral nodular discontinuous enhancement in the arterial phase and centripetal filling in the portal and delayed phases |
| continuous | lesions demonstrate rapid,uniform and intense homogeneous enhancement in the arterial phase, with a density equivalent to that of the aorta during all dynamic phases |
| circular | a complete annular enhancement zone surrounded the central low-density area |
| insignificant | without any contrast enhancement in each phase |
| delayed | contrast enhancement in the delayed phase and no enhancement in other phases |
